# Impact of continuous low water stage on the breeding environment of *Oncomelania hupensis*: a case study of Poyang Lake area in China

**DOI:** 10.1186/s40249-020-00720-4

**Published:** 2020-07-23

**Authors:** Fei Hu, Qi-Yue Li, Xiao-Feng Dai, Zhao-Jun Li, Shang-Biao Lv, Chun-Fang Lu, Yi-Feng Li, Min Yuan, Yue-Ming Liu, Ying Liu, Dan-Dan Lin

**Affiliations:** 1 Jiangxi Provincial Institute of Parasitic Diseases, No.239,First Gaoxin Rd., Gaoxin District, 330096 Nanchang, Jiangxi Province People’s Republic of China; 2 Jiangxi Province Key Laboratory of Schistosomiasis Prevention and Control, Nanchang, 330096 People’s Republic of China; 3 Jiangxi Normal University, No.99, Ziyang Avenue, Nanchang, 330022 Jiangxi province People’s Republic of China

**Keywords:** *Oncomelania hupensis*, Water level, Lake meadow, Snail control, Poyang Lake, China

## Abstract

**Background:**

*Oncomelania hupensis* is the only intermediate host of *Schistosoma japonicum* and plays a decisive role in its transmission. The variation of water level greatly affects the reproduction and growth of snails. Therefore, in this paper, we analyze the variations of water level in the Poyang Lake region from 1993 to 2016 combined with satellite imagery to elucidate the evolution of the snail breeding environment.

**Methods:**

By employing remote sensing data from 1993 to 2016 (April–June and September–November), the vegetation area of Poyang Lake and the vegetation area at different elevations were extracted and calculated. Moreover, the average daily water level data from the four hydrological stations (Hukou station, Xingzi station, Tangyin station and Kangshan station) which represent the typical state of Poyang Lake were collected from 1993 to 2016. The variance of the monthly mean water level, inundation time and the average area were analyzed by variance to find a significance level of α = 0.05.

**Results:**

According to hydrological data before and after 2003, the average water level after 2003 is significantly lower than that before 2003 in Poyang Lake. After 2003, the time of inundateing the snail breeding period was later in April to June than that before 2003, while the time of wate-falling stage in September to November moved forward after 2003 than before 2003. Of them, the lowest water level affecting the breeding and growing period of *O. hupensis* in the northern part of Poyang Lake decreased from 11 m to 9 m. After 2003, the expansion of meadow area in the north part of Poyang Lake was mainly concentrated in the elevation of 9–11 m, and the newly increased infested-meadow in the lake area was mainly concentrated in the north part of Poyang Lake.

**Conclusions:**

By comparing the change of water level characteristics in different parts of the Poyang Lake area as well as changes in meadow area before and after 2003, it is found that the water level changes mainly affect the snail breeding area in the northern part of Poyang Lake. The results are helpful for improving scientific measures for snail control in Jiangxi Province. This approach could also be applicible to Dongting Lake area and other lake areas affected by water level changes and can bring significant guidance for snail control in lake areas.

## Background

*Oncomelania hupensis* is the only intermediate host of *Schistosoma japonicum* and an essential link for schistosomiasis transmission. Snail control is therefore a key measure to prevent schistosomiasis. By the end of 2015, a standard for schistosomiasis transmission control was scheduled in China [1]. However, according to the statistics at the end of 2018, the snail breeding area in lake regions was still 3.439 billion m^2^, accounting for 94.73% of the total area in China [2]. During 2002–2010, a total of 122 million m^2^ of snail breeding regions were newly found in five provinces (Hubei, Hunan, Jiangxi, Anhui and Jiangsu) accounting for 98.30% of the total. A total of 0.59 billion m^2^ new snail areas were discovered in the lower reaches of the Yangtze River during 2009–2017, accounting for 93.70% of the total in China, mainly concentrated in lakes and marsh areas [3, 4]. At present, schistosomiasis is mainly distributed in rivers and lakes where the water level is uncontrollable, as these areas are widely distributed with snails. This is a critical concern, since there will be long-term risk of schistosomiasis transmission in the Poyang Lake and Dongting Lake regions where snails are difficult to control [5, 6].

*Oncomelania hupensis* is an amphibious species, and water is one of the essential conditions for their growth and reproduction; perennial dry areas are not suitable for snails to thrive. Young snails live in the water, and mature snails generally live on wet and food-rich land. Regions where the water level varies significantly are suitable for snail breeding if the water flow is slow, or the vegetation grows well. There are few snails found in places where the inundation time is more than 8 months or less than 1 month. The inundation time of the sparse snail breeding zone is between 6 and 8 months, and the intensive snail breeding zone areas have an inundation time of 4–5 months. Impacted by local elevation, the annual inundation and the exposure times are different, which affects the type and distribution of vegetation and thus the distribution of snails. Snails can conduct mating and spawning all year round, with a spawning period typically occurring from April to May. Snails need to spawn in moist and soft soil, and snail eggs have to be surrounded by soil before hatching [7–9]. Therefore, the variation of water level greatly affects the reproduction and growth of snails.

Jiangxi Province is seriously affected by schistosomiasis, and the Poyang Lake region is a key area of schistosomiasis control. The snail breeding area of Jiangxi has maintained at about 785–834 million m^2^ in the past decade, of which 97% is lake and marshland types, mainly in the Poyang Lake area. The Poyang Lake region is the focus of the prevention and regulation of schistosomiasis in Jiangxi Province because its extensive meadow provides favorable breeding environments for snail [2, 10]. In the mid-1980s, Jiangxi Province had measured the height difference of 615 snail infested meadows, based on the elevation of each hydrological station in the Poyang Lake area using a horizontal line of sight to investigate the distribution of snails in meadows. The snails were found to exhibit a “three-band” distribution in the elevation range of 11–16 m, namely the upper sparse region, the dense region and the lower sparse region. At that time, 94.6% of the dense snail-breeding region was distributed in the 12–15 m elevation range, as these regions are usually flooded from April to May, and then exposed from October to November. The 11–12 m and 15–16 m elevation meadows were the lower and upper sparse regions, respectively, accounting for 3.4 and 2.0% of the total snail infested meadow areas [11].

After the operation of the Three Gorges Dam in the Yangtze River began in 2003, the hydrological regime of Poyang Lake changed significantly. The daily water level comparison between 1992 and 2012 found that extremely low water level conditions (number of days below 10 m) after 2003 rose sharply from 4.34 to 24.79%, while the water level in September and October decreased by 2 m. The low water levels have appeared earlier, and the dry season was been prolonged [12]. This phenomenon was bound to lead to changes in the suitable environment for snail proliferation [13].

In view of the current changes in the ecological environment, this paper analyzes the water level changes in the Poyang Lake from 1993 to 2016 combined with analysis of remote sensing image data to clarify the evolution of the snail breeding environment, with the intention of guiding future snail population control.

## Methods

### Study area

Poyang Lake (115°49′–116°46′E and 28°24′–29°46′N) is a typical shallow lake in eastern China, located on the southern bank of the middle and lower reaches of the Yangtze River, in the north of Jiangxi Province. The water level in the lake fluctuates between a dry season (October–March) and a rainy season (April–September). The lake area in autumn and winter is less than 1000 km^2^, and the lake area can expand to more than 3000 km^2^ in summer [14, 15]. It is a seasonal lake with “winter land, summer water” hydrological characteristics. The entire terrain of the Poyang Lake catchment, from the outside and the inside, and from south to north, gradually slopes toward Poyang Lake, forming a huge basin opening to the north. The lake is usually divided by the Songmen Mountain between Duchang and Wucheng [16]. The northwest of Songmen Mountain is the northern part of Poyang Lake, a narrow and long channel that is 40 km long, 3–5 km wide, and 2.8 km at its narrowest. The southeast of Songmen Mountain is the southern part of Poyang Lake, and consists of the main body of the lake, 133 km long and 74 km wide at its widest point.

### Water level data

Four hydrological stations representing the typical situation of Poyang Lake were selected, including Hukou Station in the lower reaches of the northern part of Poyang Lake, Xingzi Station in the upper reaches of the northern part of Poyang Lake, Tangyin Station in the middle of the main lake of Poyang Lake, and Kangshan Station in the upper reaches of the southern part of Poyang Lake. The daily average water level data from each hydrological site from 1993 to 2016 was collected.

### Processing of elevation data [17]

Using a topographic map of the Poyang Lake bottom in 2009, digital elevation model (DEM) grids were projected into the WGS-84 coordinate system and the Transverse Mercator projection using the ArcGIS software R10.5 (ESRI, RedLands, USA). The DEM data was preprocessed before interpolation, which includes separating the conglutinated contour lines and connecting the disconnected contour lines, establishing the elevation index table concurrently, and then calculating the elevation data interpolation. The method of contour raster image profile interpolation was used for elevation data interpolation in contour line rendering. The elevation accuracy was evaluated by the checkpoint method and contour playback methodology.

### Remote sensing image screening

As Poyang Lake is covered by one Landsat scene (Path 121 Row 040), a total of 47 thematic mapper (TM) and operational land imager (OLI; 30 m) between 1993 and 2016 were derived from the LIT level product of TM sensor and OLI sensor ESPA-LSRD (https://espa.cr.usgs.gov/). Since April to June is the typical breeding time for snails, Landsat-4/5 and Landsat-8 remote sensing images of Poyang Lake area from April to June and September to November of each year were obtained, as well as Landsat-7 data from 2012. In order to fully determine the scope of the meadow region in Poyang Lake, no less than one scene image was selected in April–June or September–November. The final images had a total of 44 scenes covering 17 years including 1993–1997, 1999–2004, 2006, 2007, and 2013–2016 (Table 1).

**Table 1:** Remote sensing images used during 1993–2016

No.	Data identification	Cloud (%)	Date	No.	Data identification	Cloud (%)	Date
1	LT51210401993111BJC00	36.00	21 Apr 1993	25	LT51210402004286BKT02	2.00	12 Oct 2004
2	LT51210401993159CLT00	25.00	8 Jun 1993	26	LT51210402005272BJC02	1.00	29 Sep 2005
3	LT51210401993319BJC00	0.00	15 Nov 1993	27	LT51210402006147BJC00	26.00	27 May 2006
4	LT51210401994306BJC00	0.00	2 Nov 1994	28	LT51210402006307BJC01	0.00	3 Nov 2006
5	LT51210401995245CLT00	3.00	2 Sep 1995	29	LT51210402007102BJC00	53.00	12 Apr 2007
6	LT51210402008137BKT00	15.00	5 Nov 1995	30	LT51210402007278BKT01	0.00	5 Oct 2007
7	LT51210401996328CLT00	2.00	23 Nov 1996	31	LT51210402008137BKT00^a^	2.00	16 May 2008
8	LT51210401997122BJC00	7.00	2 May 1997	32	LC81210402013134LGN03	6.09	14 May 2013
9	LT51210401997250BJC00	3.00	7 Sep 1997	33	LC81210402013278LGN01	0.02	5 Oct 2013
10	LT51210401998093BJC00^a^	0.00	3 Apr 1998	34	LC81210402013294LGN01	12.96	21 Oct 2013
11	LT51210401999096BJC00	0.00	6 Apr 1999	35	LC81210402013326LGN01	1.60	6 Nov 2013
12	LT51210401999320BJC00	7.00	16 Nov 1999	36	LC81210402013326LGN01	1.60	22 Nov 2013
13	LT51210402000131BJC00	1.00	10 May 2000	37	LC81210402014121LGN01	0.13	1 May 2014
14	LT51210402000307BJC00	0.00	2 Nov 2000	38	LC81210402014153LGN01	17.20	2 Jun 2014
15	LT51210402001181BJC02	1.00	30 Jun 2001	39	LC81210402014249LGN01	21.72	6 Sep 2014
16	LT51410402000287BKT00	3.00	18 Sep 2001	40	LC81210402014281LGN00	0.27	8 Oct 2014
17	LT51210402001293BJC00	0.00	20 Oct 2001	41	LC81210402014297LGN01	4.03	24 Oct 2014
18	LT51210402001325BJC00	2.00	21 Nov 2001	42	LC81210402015140LGN01	60.53	20 May 2015
19	LT51210402002280BJC00	0.00	7 Oct 2002	43	LC81210402015156LGN01	41.04	5 Jun 2015
20	LT51210402002312BJC00	1.00	8 Nov 2002	44	LC81210402015252LGN01	1.45	9 Sep 2015
21	LT51210402003267BJC00	0.00	24 Sep 2003	45	LC81210402015284LGN01	0.62	11 Oct 2015
22	LT51210402003283BJC00	4.00	10 Oct 2003	46	LC81210402016175LGN00	1.63	23 Jun 2016
23	LT51210402004126BJC00	1.00	5 May 2004	47	LC81210402016271LGN00	0.34	27 Sep 2016
24	LT51210402004270BJC00	5.00	26 Sep 2004				

^a^Abandoned

### Data processing

Image preprocessing consisted of two steps: band fusion and image clipping. Bands 4, 5, and 6 were selected for band fusion to obtain a base map of Poyang Lake. Using ArcGIS, the merged image and the simultaneous normalized differential vegetation index (NDVI) data were clipped using the Poyang Lake boundary vector data, and then outputted in TIF format.

Object-oriented classification technology collected adjacent pixel recognition spectral elements, and made full use of the spatial, texture and spectral information of high-resolution full-color and multi-spectral data to segment and classify features. Vegetation areas were extracted from 44 scene images using the Cognition Developer platform [18]. In order to reduce the error of the output data, the multi-scale segmentation fixed parameter value is set as 300. According to the actual situation of each scene image, the set threshold value was estimated, and the image map of the marked vegetation distribution of Poyang Lake area was imported into the ArcGIS platform to fuse vegetation images of the same year. The vegetation area of Poyang Lake was calculated, and the elevation data was superimposed to extract the vegetation area in different elevations, which makes analysis of the output data by SPSSR 20.0 (IBM, Armonk, USA) platform more convenient.

### Data analysis

We compare and analyzes various kinds of data before 2003 (1993–2003) and after 2003 (2004–2016), since this year was when the operation of the Three Gorges Dam began. The variance of monthly mean water level, the number of flooded days and the average area were analyzed by variance at a test level of α = 0.05. All statistical analyses were performed using SPSS R20.0 (IBM, Armonk, USA) software.

## Results

### Average water level change

**Fig. 1:**
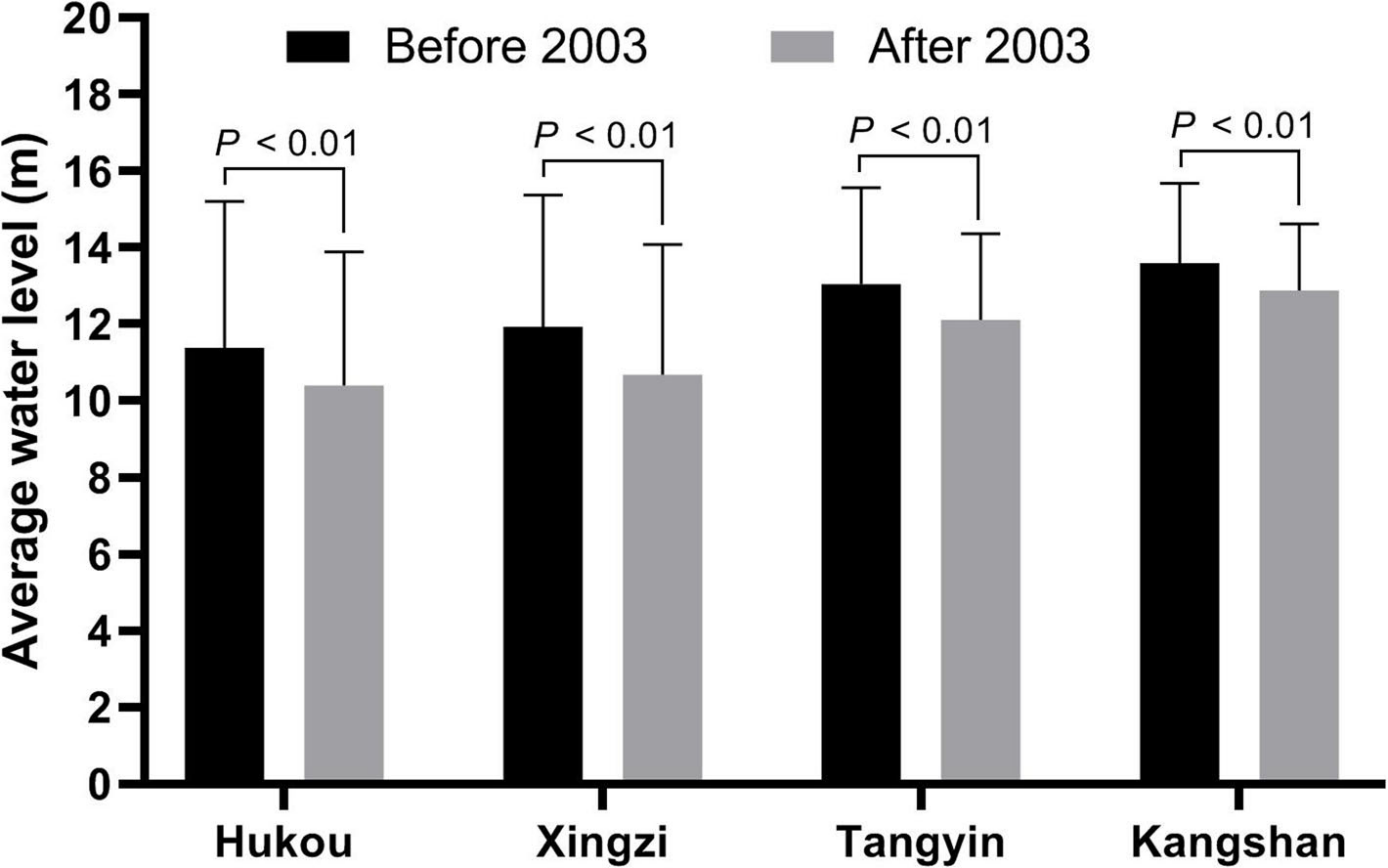
Comparison of the average water level of four hydrological stations in Poyang Lake ordered from north to south before and after 2003

The average water levels at Hukou Station, Xingzi Station, Tangyin Station and Kangshan Station before 2003 were 11.39 ± 3.82 m, 11.93 ± 3.44 m, 13.04 ± 2.52 m and 13.59 ± 2.09 m, respectively. The average water levels at the four hydrological stations after 2003 were 10.40 ± 3.48 m, 10.69 ± 3.39 m, 12.11 ± 2.25 m and 12.87 ± 1.74 m, respectively. The average water levels at the four hydrological stations after 2003 were thus significantly lower than those before 2003 (*P*_*all*_ < 0.01) (Fig. 1).

### Effect of water level variations on the breeding period of snails

The main reproduction period of the *Oncomelania hupensis* is from April to June. The daily water level changes from April to June before and after 2003 at the Kangshan Station (southern part of Poyang Lake) and the Hukou Station (northern part of Poyang Lake) were selected to be analyzed and compared. The daily water level of Hukou Station in the northern part of Poyang Lake revealed that the average water level in April before 2003 was 8.83–11.74 m, and the water level fluctuated mainly between 10 and 11 m. The average water level in May was 9.50–15.37 m, and the water level exceeded 11 m in most years. The average water level in June was 12.74–16.53 m, completely inundating the 11 m water level line. After 2003, the average water level in April was 7.72–12.75 m, mainly fluctuating around 9 m. The average water level in May was between 7.42 m and 15.49 m, and mainly fluctuated around 11 m. The average water level in June was 11.77 m to 16.05 m, and the 11 m water level line was completely submerged.

Since 11 m is the minimum water level reference of the snail breeding period, we consider the occurrence of when this level is first reached in each year at the start of the rainy season (Table 2). The water level exceeded 11 m in April in most years before 2003, though in 1993 and 2000, the water level reached 11 m in May. After 2003, there was 5 years where the water level reached 11 m in May, 3 years when the 11 m level was only reached in June. The average number of inundation days above 11 m before and after 2003, were 10 d and 6 d, respectively, at a high level of significance (F = 4.76, *P* < 0.05). We also consider that the water level reached 9 m in April every year before 2003, However, after 2003, the 9 m level was first reached in May for 3 years, and 1 year in which it was postponed to June (Table 2).

**Table 2:** Daily water level changes at Hukou Station in the northern part of Poyang Lake from April to June during 1993–2016

Year	Monthly mean water level			Water level (9 m)						Water level (11 m)					
				April		May		June		April		May		June	
	April	May	June	Rising date	Rising days	Rising date	Rising days	Rising date	Rising days	Rising date	Rising days	Rising date	Rising days	Rising date	Rising days
1993	9.30	11.99	13.75	1	28		31		30	–	0	8	24		30
1994	10.54	12.79	14.76	7	24		31		30	15	16		31		30
1995	10.43	13.06	16.52	10	21		31		30	19	12		31		30
1996	10.83	10.66	13.93	1	30		31		30	7	15	19	13		30
1997	10.79	11.79	12.74	3	28		31		30	21	10		31		30
1998	11.74	12.59	14.99	1	30		31		30	1	29	10	22		30
1999	8.83	12.72	14.56	19	12		31		30	23	8		31		30
2000	9.56	9.50	13.47	12	19		18		30	–	0	2	3	5	26
2001	9.69	12.65	12.82	10	21		31		30	27	4		29	10	21
2002	9.58	15.37	14.99	20	11		31		30	25	6		31		30
2003	10.40	13.43	13.37	1	30		31		30	17	14		31		30
2004	8.03	11.74	13.28	–	0	2	29		30	–	0	12	20		30
2005	8.79	11.64	15.17	17	5	7	25		30	–	0	13	19		30
2006	10.04	11.86	13.74	1	20		31		30	17	9	11	21		30
2007	7.72	8.69	11.77	–	0	1	13	2	29	–	0	–	0	14	17
2008	9.80	10.02	12.49	6	25		31		30	–	0	–	0	11	20
2009	9.32	11.82	12.82	20	11		31		30	25	6		31		30
2010	11.29	13.78	15.85	10	21		31		30	15	16		31		30
2011	7.19	7.42	12.16	–	0	–	0	8	23	–	0	–	0	12	19
2012	9.60	14.27	15.52	16	15		31		30	27	4		31		30
2013	10.19	12.42	14.27	1	30		31		30	10	2	10	22		30
2014	9.06	12.45	13.88	19	12		31		30	30	1		31		30
2015	10.59	11.79	16.05	4	27		31		30	7	12	14	17		30
2016	12.75	15.49	15.97	1	30		31		30	8	23		31		30

- Water level does not exceed 9 m or 11 m in the current month

**Fig. 2:**
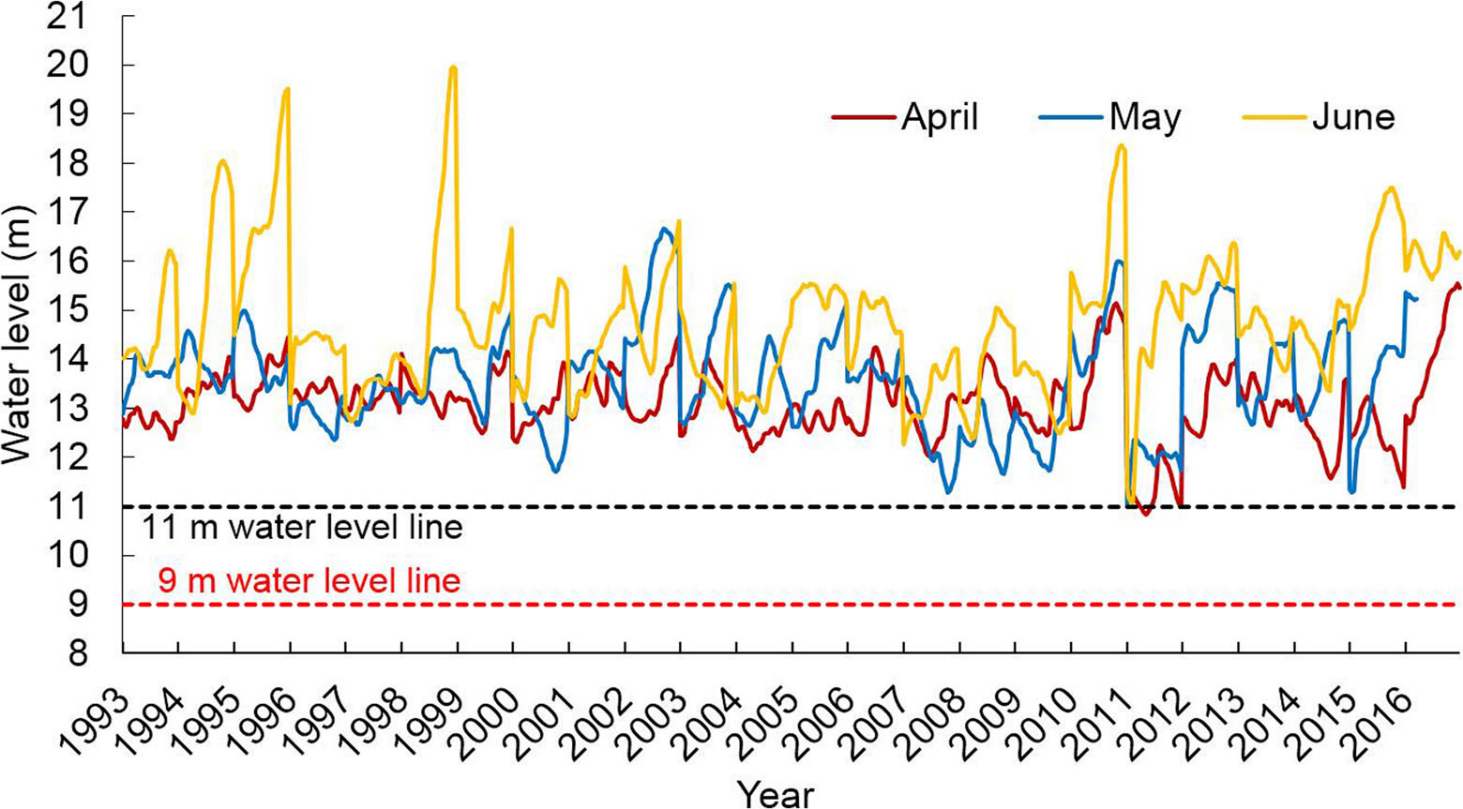
Daily water level changes at Kangshan Station in the southern part of Poyang Lake from April to June during 1993–2016

The daily water level of Kangshan Station in the southern part of Poyang Lake illustrates that during April to June of 2013–2016, the water level was always above 11 m, except in April 2011, when the water level was below 11 m for 6 days (Fig. 2).

### Effect of water level variations on the growth period of snails

The data from Hukou Station in northern part of Poyang Lake showed that the water level below 11 m was reached in October–November before 2003; there were 4 years when the water level dropped below 11 m in October, 6 years in November, and only 1 year (1997) in September. After 2003, there are 10 years in which the water level declined to less than 11 m in October, and 4 years when it dropped below 11 m in September, and no occurrences in November. Before 2003, during September–November the number of days of water level below 11 m was 5–30 d, with an average of 9 d. After 2003, it was 5–31 d, with an average of 18 d. The latter is significantly higher than the former (F = 9.74, *P* < 0.01).

Before 2003, the average water level during September, October and November was 11.70–18.38 m, 10.31–13.79 m, and 7.63–12.25 m, respectively. After 2003, the average water level was 8.54–15.42 m, 7.19–12.20 m, and 6.36–11.59 m in September, October and November, respectively (Table 3). Before 2003, the water level dropped to below 9 m in November of each year, but after 2003, there were 6 years in which this level was reached earlier in October, and 3 years in which it reached in September.

**Table 3:** Daily water level changes at Hukou station in the northern part of Poyang Lake from September to November during 1993–2016

Year	Monthly mean water level			Water level (9 m)					Water level (11 m)						
				September		October		November	September			October		November	
	September	October	November	Falling date	Falling days	Falling date	Falling days	Falling date	Falling days	Falling date	Falling days	Falling date	Falling days	Falling date	Falling days
1993	16.96	13.66	11.27	–	0	–	0	–	0	–	0	–	0	18	7
1994	12.47	12.62	9.38	–	0	–	0	13	18	–	0	–	0	7	24
1995	13.07	11.85	9.04	–	0	–	0	13	18	–	0	–	0	5	26
1996	14.72	11.58	10.36	–	0	–	0	4	5	–	0	24	8		19
1997	11.70	11.18	8.55	–	0	–	0	7	22	20	11		10		30
1998	18.38	13.79	8.84	–	0	–	0	14	17	–	0	–	0	3	28
1999	17.33	13.56	10.83	–	0	–	0	–	0	–	0	–	0	15	16
2000	14.59	13.74	12.25	–	0	–	0	–	0	–	0	–	0	23	8
2001	13.19	11.78	9.62	–	0	–	0	23	8	–	0	23	9		30
2002	14.94	10.31	10.57	–	0	–	0	–	0	–	0	6	26		22
2003	14.13	12.37	7.63	–	0	–	0	3	28	–	0	27	5		30
2004	13.88	11.29	8.42	–	0	–	0	2	29	–	0	20	11		30
2005	15.42	12.20	10.14	–	0	–	0	30	1	–	0	28	4		30
2006	8.54	7.19	6.86	1	22		31		30	1	30		31		30
2007	14.32	10.40	7.03	–	0	23	9		30	–	0	13	19		30
2008	15.00	10.81	11.59	–	0	26	6		5	–	0	16	16		8
2009	12.42	7.77	6.68	–	0	7	25		30	26	5		31		30
2010	14.77	11.96	8.08	–	0	–	0	10	21	–	0	26	6		30
2011	9.67	8.74	8.40	9	10	11	21		21	1	25		30		30
2012	13.74	10.89	9.31	–	0	–	0	1	13	–	0	12	20		30
2013	10.27	8.95	6.36	–	0	16	16		30	5	25	10	22		30
2014	14.47	11.50	9.62	–	0	26	6	22	9	–	0	19	13		28
2015	11.63	10.62	10.47	–	0	28	4		6	–	0	17	15		17
2016	10.42	8.12	9.14	23	8		31		30	11	20		31		30

- Water level does not exceed 9 m or 11 m in the current month

**Fig. 3:**
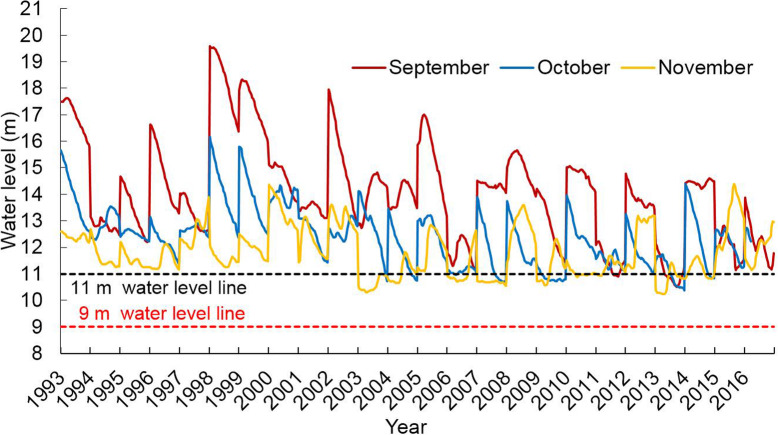
Daily water level changes at Kangshan Station the southern part of Poyang Lake from September to November during 1993–2016

The daily water level at Kangshan Station in the southern part of Poyang Lake shows that the water level in September–November was basically at or above 11 m during most of 1993–2016, and the water level was above 9 m for the entire year (Fig. 3).

### Inter-annual evolution of the snail breeding environment

The meadow area of Poyang Lake area extracted by remote sensing images was 957.81–2278.55 km^2^ from 1993 to 2016. A single-sample *t*-test showed that the inter-annual meadow area changed significantly (*t* = 16.984, *P* < 0.01). The meadow ratio of in northern Poyang Lake was between 11.38 and 27.12%. The comparison of different elevation groups reveals that the change of meadow areas in the south and north of Poyang Lake were mainly found in the elevation range of 9–16 m influenced by water level change (F_A_ = 12.186, *P*_A_ < 0.01; F_B_ = 8.219, *P*_B_ < 0.05; F_C_ = 10.72, *P*_C_ < 0.01; F_D_ = 7.677, *P*_D_ < 0.05) (Table 4).

**Table 4:** Distribution of meadow areas in different areas and elevations of Poyang Lake during 1993–2016

Years	South (km^2^)					North (km^2^)					Total
	≤ 9 m	> 9–≤ 11 m ^A^	> 11–≤116 m^B^	> 16 m	Subtotal	≤ 9 m	> 9–≤ 11 m ^C^	> 11–≤ 16 m^D^	> 16 m	Subtotal	
1993	4.38	11.19	1127.59	85.30	1228.47	3.88	15.97	187.20	48.55	255.60	1484.07
1994	1.61	3.53	789.67	54.04	848.85	0.64	1.21	85.02	22.08	108.95	957.81
1995	3.72	13.28	1334.91	85.37	1437.29	5.74	15.96	209.34	49.40	280.44	1717.72
1996	2.50	9.78	1222.16	76.58	1311.02	1.53	9.94	176.82	40.35	228.64	1539.67
1997	3.64	8.88	1002.01	85.01	1099.54	3.58	5.21	142.73	48.89	200.41	1299.94
1999	3.76	12.42	1207.28	85.10	1308.56	17.66	74.48	219.39	48.64	360.16	1668.72
2000	2.56	7.66	963.01	84.16	1057.39	3.41	12.95	167.29	48.58	232.23	1289.62
2001	8.12	65.33	1427.81	86.07	1587.34	12.32	95.62	239.86	49.54	397.33	1984.67
2002	3.05	7.95	977.50	84.06	1072.55	2.45	12.62	167.18	46.98	229.22	1301.78
2003	2.34	6.05	761.76	85.15	855.31	2.67	2.30	82.71	47.84	135.51	990.82
2004	5.10	13.73	1304.76	85.77	1409.36	5.54	41.20	200.76	49.01	296.52	1705.88
2006	6.07	92.03	1483.39	85.65	1667.14	29.75	131.24	251.27	48.46	460.71	2127.85
2007	14.67	80.71	1479.12	86.02	1660.52	98.95	227.10	242.53	49.46	618.04	2278.55
2013	10.18	167.92	1533.61	84.65	1796.36	18.15	167.20	241.89	42.43	469.67	2266.03
2014	2.67	71.03	1368.84	85.92	1528.46	1.28	65.00	233.81	47.08	347.17	1875.63
2015	2.13	12.21	1181.92	84.22	1280.48	0.86	12.69	206.71	44.22	264.48	1544.95
2016	2.47	122.37	1156.38	83.71	1364.93	5.11	140.98	205.82	48.29	400.20	1765.13

**Fig. 4:**
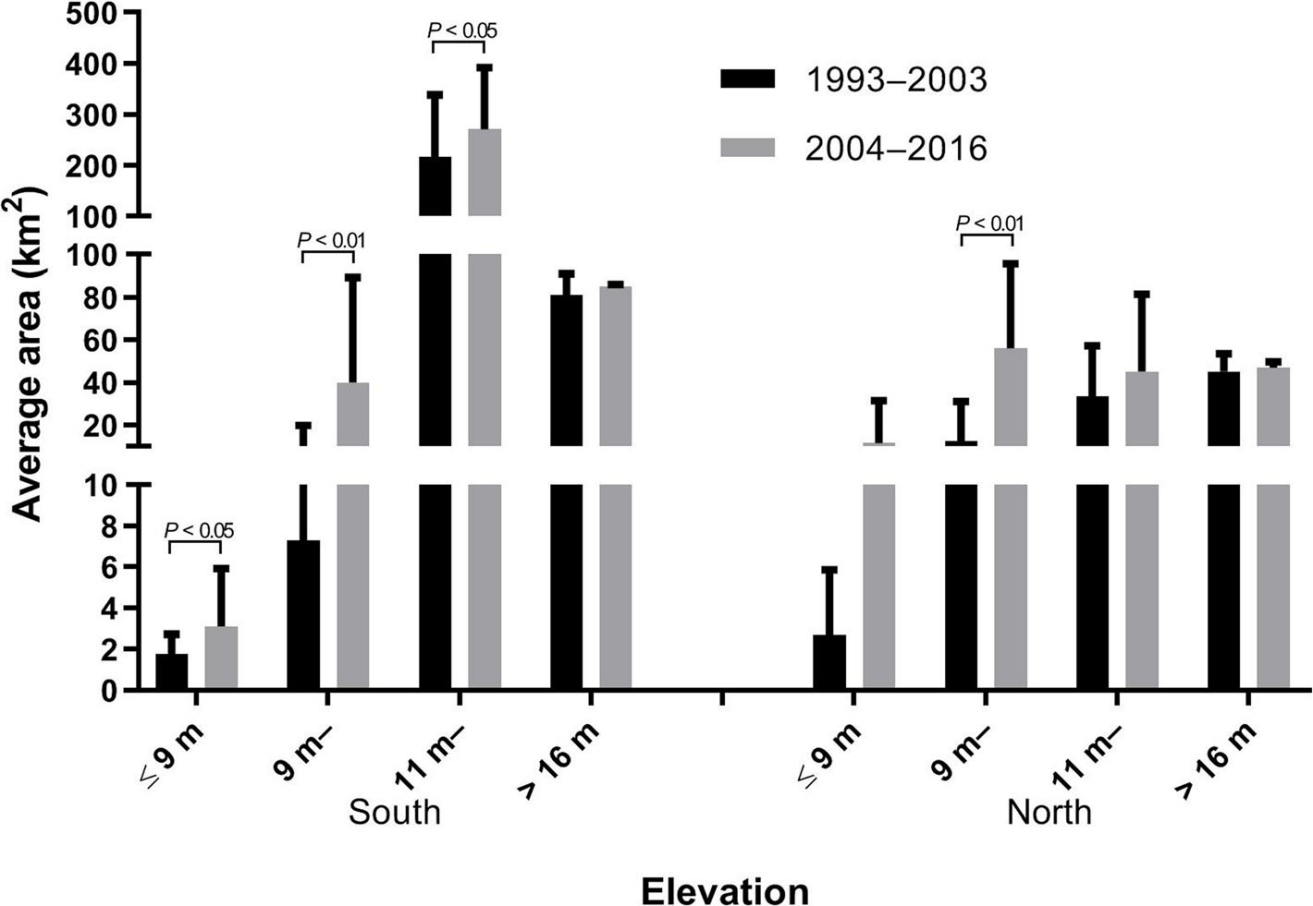
Average area of snail infested meadows in Poyang Lake area before and after 2003

We again took 2003 as the demarcation point to compare the average meadow area of different elevations in the southern and northern parts of Poyang Lake. The results revealed that the average meadow area in the southern part of Poyang Lake after 2003 was significantly higher (*P* < 0.05) than that before 2003 in three elevation ranges (≤ 9 m, > 9–≤ 11 m, and 11 m), while in the northern part, only the 9–11 m elevation range meadow area after 2003 was significantly higher than that before 2003 (*P* < 0.01) (Fig. 4).

### Inter-annual change of newly increased snail infested meadows

Through a retrospective survey, a total of 80 new parcels of snail infested meadows were found in the Poyang Lake area from 1993 to 2016, with an aggregate area of 9993.96 ha. In the southern part of Poyang Lake, 22 snail infested meadows appeared newly before 2003, with a total area of 1345.14 ha. However, only 4 snail infested meadows appeared newly after 2003. In the northern part of Poyang Lake, 19 parcels of snail infested meadows were newly increased before 2003, with a total area of 3573.67 ha, and 35 parcels of snail infested meadows were newly increased after 2003. Further analysis of the distribution of newly increased snail infested meadows from 1993 to 2016 reveal that before 2003, newly increased snail infested meadows emerged in both the south and north part of Poyang Lake was almost same, and mainly occurred in 2002. After 2003, most of the newly increased snail infested meadows mainly distributed in the northern part of Poyang Lake and mainly emerged in 2016 (Table 5).

**Table 5:** Newly increased snail infested meadows regions in the Poyang Lake area during 1993–2016

Year	Southern		Northern	
	Numbers	Area (ha)	Numbers	Area (ha)
1998	1	19.98	0	
2000	0		1	58.13
2001	5	244.55	2	129.71
2002	15	1011.91	16	3385.83
2003	1	68.70	0	
2004	0		1	11.20
2008	0		1	0.10
2009	0		1	7.67
2010	1	141.00	0	
2013	1	3.87	5	99.48
2014	0		2	5.49
2015	1	27.30	0	
2016	1	27.30	25	4751.74

## Discussion

Previous studies have shown that *Oncomelania hupensis* normally distribute in elevations ranging between 11 and 16 m in the Poyang Lake area, with 11 m typically indicating the lower limit of snail breeding [11]. The main spawning period of snails each year is from April to May. Only when the soil is kept moist can they adapt to spawning. The results of water level changes from 1993 to 2016 show that the average water level after the 2003 is about 1 m lower than before 2003. In the northern part of Poyang Lake before 2003, the water level during April–May was about 11 m during most years but it was lower, around 9 m, after 2003. However, in the southern part of Poyang Lake from 1993 to 2016, the water level during April–June changed only negligibly (being approximately 11 m in all years).

The change of water level indicates that in northern Poyang Lake, the breeding and living area of snails will change in order to adapt to the changing environment, while in the southern part, the breeding environment will remain consistent, since the water levels have not changed significantly. The change in water level further confirms that in northern Poyang Lake, the reproduction and survival area of snails will transform in the 9–11 m elevation range due to the changes in ecological characteristics. However, the southern lake should not expect to see any significant changes in snail population dynamics, as the lower limit elevation been maintained consistently [17]. Additionally, the water level changes in northern Poyang Lake from September to November suggested that after 2003, the meadows below 11 m elevation have been exposed earlier due to an advanced dry season, and water levels have remained at a lower level.

The water resources in the middle and lower reaches of the Yangtze River have changed significantly, especially in the dry season. The water coming from the main stream of the Yangtze River in the dry season has decreased, which has accelerated water discharge from Poyang Lake, resulting in a decline of water levels and a reduction of wetland areas.

The meadow area has increased with the decrease of water level, and the distribution of vegetation reveals that the trend of distribution has transferred towards the lower elevation and the central lake basin [19], resulting in a gradual emergence of the lake bottom and formed meadow with carex, *Triarrhena lutarioriparia*, which is suitable for increased snail breeding [20, 21]. Furthermore, these areas more easily allow a high risk environment for *Schistosoma* infection due to human and animal activities.

The image analysis of the grassland area further confirmed these results. The average meadow area in southern Poyang Lake at an elevation range of 16 m or below before 2003 was significantly lower than that after 2003. When combined with the change of water level in the southern part of the lake and the relationship between the snails breeding period and water level, it is suggested that the snails will not increase breeding in the southern like since the water level has maintained above 11 m. In northern Poyang Lake after 2003, the areas above 9 m that is suitable for snail to lay their eggs increased significantly as compared to before 2003. This can also be confirmed from field investigation results [17].

Using remote sensing and GIS technology combined with snail population data in the lake area to determine environmental factors closely related to snail breeding, previous studies selected the Poyang Lake National Nature Reserve in the northwest of the main lake area of Poyang Lake to monitor the dynamic change of the distribution pattern of snail breeding regions from October 2016 to July 2017. These results further verified that the distribution of snails presented an expansion trend to the low elevation zones because of the exposed shoals, a prolonged exposure time, and an increase of vegetation exposure under the influence of longer-term low water levels. It is from these previous efforts also suggested that it is more reasonable to set an 8.5–11 m elevation range as the ideal snail proliferation zone [22].

After the operation of Three Gorges Dam, water levels in Poyang Lake and Dongting Lake have changed due to variations of the Yangtze River hydrological state. Previous studies analyzed the favorable factors for the control of schistosomiasis from the perspective of schistosomiasis morbidity, snail density decline and extinction [23–25]. However, other factors, such as mud beach turning into meadows, new snail habitat creation, etc., have not yet been considered, which may further aggravate the schistosomiasis epidemic.

Another recent study suggested that the time of inundation and the establishment of the Three Gorges Dam might not be the direct cause to the natural extinction of snails, though it may change the distribution of snails in the East Dongting Lake. This was determined through comparing the elevation, water level data, and the number of inundation days before and after the Three Gorges Dam operation in two meadows: a nature snail extinction meadow and a meadow with snails [26]. Yet another approach involved analyzing snail data during the period of 2004–2014 combined with elevation data in the Dongting Lake area. Their results showed that the snail density continued to decrease in the middle and high elevation areas, while in the low elevation, snail density began to decline in 2003, but then rose gradually after 2011 and reached stabilization after 2017 [27].

These previous results suggest that long-term occurrence of low water levels will increase the spread of schistosomiasis. For example, in Hukou County of Jiangxi Province, a batch of acute schistosomiasis outbreaks occurred in 2013 [28]. No epidemic situation monitoring was carried out in the locations of acute infection due to long term flooding and occasionally exposed mudflats. However, the original mudflats gradually became meadows and suitable for snail breeding due to the continuous low water level and the extension of dry season after 2003. A retrospective survey of snail populations revealed that 41 parcels of snail infested meadows were newly increased in the Poyang Lake area from 1993 to 2003 before the operation of Three Gorges Dam, and 22 and 19 parcels increased in the south and north of Poyang Lake, respectively. The occurrence of new parcels may be related to the accumulation of silt in the lake area. There were a total 39 parcels of newly increased snail infested meadows after 2004, and 35 parcels (accounting for 89.74%) were distributed in the north of Poyang Lake, which further illustrated that the variation in water level may lead to a positive feedback of the expansion of snail breeding environments in the northern Poyang Lake area. Since this retrospective survey could not complete the collection of snail density data in the snail breeding environment, the interaction between the snail density and water level variation needs further research.

## Conclusions

We found that the water level changes mainly affect the snail breeding area in the north part of Poyang Lake by comparing the change of water level characteristics of Poyang Lake from the perspective of different parts of Poyang Lake area as well as changes in meadow area before and after 2003. The results are helpful to make more scientific control measures for snail control in Jiangxi Province. This approach could also be applicible to Dongting Lake area and other lake areas affected by water level changes and can bring significant guidance for snail control in lake areas.

## Data Availability

The dataset used in the study are available from the corresponding author on reasonable request.

## Abbreviations

DEM: Digital elevation model
TM: Thematic mapper
OLI: Operational land imager
NDVI: Normalized differential vegetation index
